# Simulation of Fluid-Structure and Fluid-Mediated Structure-Structure Interactions in Stokes Regime Using Immersed Boundary Method

**DOI:** 10.1155/2014/782534

**Published:** 2014-02-23

**Authors:** Masoud Baghalnezhad, Abdolrahman Dadvand, Iraj Mirzaee

**Affiliations:** ^1^Department of Mechanical Engineering, Urmia University of Technology, Urmia 57166 93187, Iran; ^2^Department of Mechanical Engineering, Urmia University, Urmia 57561 51818, Iran

## Abstract

The Stokes flow induced by the motion of an elastic massless filament immersed in a two-dimensional fluid is studied. Initially, the filament is deviated from its equilibrium state and the fluid is at rest. The filament will induce fluid motion while returning to its equilibrium state. Two different test cases are examined. In both cases, the motion of a fixed-end massless filament induces the fluid motion inside a square domain. However, in the second test case, a deformable circular string is placed in the square domain and its interaction with the Stokes flow induced by the filament motion is studied. The interaction between the fluid and deformable body/bodies can become very complicated from the computational point of view. An immersed boundary method is used in the present study. In order to substantiate the accuracy of the numerical method employed, the simulated results associated with the Stokes flow induced by the motion of an extending star string are compared well with those obtained by the immersed interface method. The results show the ability and accuracy of the IBM method in solving the complicated fluid-structure and fluid-mediated structure-structure interaction problems happening in a wide variety of engineering and biological systems.

## 1. Introduction

There are abundant scientific and engineering applications where a flexible structure is immersed in a viscous incompressible fluid. The motion of the structure can induce the fluid flow motion and vice versa. The combination of the fluid and structure motions constitutes the so-called fluid-structure interaction (FSI) problem (see [1, 2] for examples). The immersed boundary method (IBM) is an efficient procedure for modeling and simulating FSI problems especially when an elastic structure interacts with a fluid.

Peskin [3] was the first who introduced the IBM method to simulate the blood flow in heart valves assuming a very low Reynolds number and two-dimensional flow. Peskin and McQueen [4] developed the IBM to simulate three-dimensional heart flows. The key idea of this method is to represent the effect of structure on the fluid flow by a singular force added to the Navier-Stokes equations to mimic the no-slip condition on the structure. The IBM uses a mixture of Eulerian and Lagrangian variables, which are connected by a smoothed approximation of the Dirac delta function. For the numerical implementation the Eulerian variables are defined on a fixed Cartesian mesh, while the Lagrangian variables representing the immersed boundary are defined on a curvilinear grid that lies on top of the fixed Cartesian mesh. For immersed flexible boundaries, the Lagrangian forcing can be derived by the principle of virtual work [5]. The IBM has been used to simulate a number of problems including the swimming of eels, sperm, and bacteria [6–8], ameboid deformation [9], platelet aggregation during blood clotting [9, 10], and the deformation of red blood cells in a shear flow [11].

Our interest in IBM is motivated by the ability of this method in simulating a wide range of complex physical phenomena, which share the essential difficulties of the problems considered in the present work. The focus is mainly on the case of very low Reynolds number because many interesting applications occur in this regime. In the present work, the Stokes equations are solved in the presence of immersed boundaries on an unbounded domain. A number of researchers have used the IBM method to simulate the cellular and subcellular biological processes that occur at a very low Reynolds number [6, 12–14]. Many such applications include objects that could be represented as slender bodies or particles immersed in a fluid. Among the slender bodies, one can refer to the tails of spermatozoa, eukaryotic cilia, bacterial flagella, microtubules, chromosomes, and strands of DNA and RNA. In addition, the cells, cell organelles, and individual protein molecules might roughly be represented as spherical particles. At larger scales, fibers are the basic constituents of many biological tissues, and many IBM computations involve elastic structures that are constructed of fibers [5]. Apart from biology, the IBM has been used to study the particle and filament suspensions at low Reynolds number flows [15–17]. Slender bodies in the IBM method have often been represented by a linear array of points instead of using a two- or three-dimensional mesh of Lagrangian immersed boundary points to define the position of the body [8, 14, 18].

In the present work, the immersed boundary-induced Stokes flow is studied using IBM. Unlike most of the previous studies, the fluid in the present work is initially at rest. The motion of the immersed boundaries induces flow motion, which lies in the Stokes flow regime. The following three test cases are considered: (i) Stokes flow induced by motion of an extending star string (FSI); (ii) Stokes flow induced by motion of a filament fixed at one end (FSI); (iii) Stokes flow induced by motion of the same filament considered in the second test case, but now deforming a secondary elastic immersed boundary placed in the flow domain (fluid-mediated structure-structure interaction). In order to benchmark the numerical simulation carried out in the present work, the results of the first test case are compared with those obtained by the immersed interface method [19]. The last two test cases are found in many biological and engineering applications, and are studied for the first time in the present work using IBM. In all of the cases studied in the present work, the flexible structure is considered massless.

## 2. Mathematical Formulation

### 2.1. Governing Equations

The flow induced by the elastic boundary motion is assumed to be in the Stokes regime and hence it is governed by the Stokes equations. The Stokes flow refers to the highly viscous fluid flow, in the limit where the Reynolds number tends to zero and both the inertial and convection terms are omitted from the Navier-Stokes equations [20–22]. In the immersed boundary method, the Stokes equations for two-dimensional problems can be written as

(1) $$\begin{array}{c} \nabla \cdot \vec{V}=0, \end{array}$$

(2) $$\begin{array}{c} 0=-\frac{\partial p}{\partial x}+\mu \left(\nabla^{2}u\right)+f_{x}, \end{array}$$

(3) $$\begin{array}{c} 0=-\frac{\partial p}{\partial y}+\mu \left(\nabla^{2}v\right)+f_{y}, \end{array}$$

where ∇^2^ denotes the Laplacian, $\vec{V}(u,v)$ is the velocity vector, *p*  is the pressure, and *μ* is the viscosity. It may be noted that $\vec{f}(f_{x},f_{y})$ is the force density vector applied to enforce the no-slip boundary condition along the structure interface and is not the gravitational force vector. Since there is no inertia in the system, the time evolution of the flow is governed by the time dependence of the forces $\overset{\to }{f}$. Once $\overset{\to }{f}$ is known at a given instant of time, then (1)–(3) are elliptic and their solution is independent of the time history of the flow.

Equations (1)–(3) can either be solved as a coupled system or be reduced to three separate Poisson problems. In the latter case, for instance, (2) and (3) can be differentiated, respectively, with respect to *x* and *y* and be added together to give the following relation:

(4) $$\begin{array}{c} \nabla^{2}p=\vec{\nabla }\cdot \vec{f}. \end{array}$$

Since the right-hand side of (4) is known, it is a Poisson equation, which is to be solved for pressure. Once *p* is known, (2) and (3) are independent Poisson problems for *u* and *v*, respectively.

It is worth mentioning that the density force $\vec{f}$ is nonzero only on the immersed boundary and is zero elsewhere in the fluid domain. In order to calculate $\vec{f}$, which arises from the elastic energy of the immersed boundary, we need to specify the material points of the boundary. Thus, we need a Lagrangian description of the boundary. Suppose that $\vec{X}(s,t)$ is an arbitrary point on the immersed boundary where the parameter *s*  (0 ≤ *s* ≤ *l*_0_, where *l*_0_ is the length of the immersed boundary at equilibrium) indicates the Lagrangian coordinate along the boundary and *t* denotes time. According to the principle of virtual work, the Lagrangian force $\vec{F}$ is derived using the variational derivative of the elastic energy functional $E[\vec{X}(s,t)]$; that is,

(5) $$\begin{array}{c} \vec{F}=-\frac{\partial E\left(\vec{X}\right)}{\partial \vec{X}}. \end{array}$$

The elastic energy $E(\vec{X})$ consists of the stretching and bending energies [23]:

(6) $$\begin{array}{c} E\left[\vec{X}\right]=\frac{1}{2}c_{s}\int {\left(\left|\frac{\partial \vec{X}}{\partial s}\right|-1\right)}^{2}ds+\frac{1}{2}c_{b}{\int \left|\frac{\partial^{2}\vec{X}}{\partial s^{2}}\right|}^{2}ds, \end{array}$$

where *c*_*s*_ and *c*_*b*_ are constants denoting the stiffness and bending coefficients, respectively. After substitution of (6) into (5) and doing some mathematical operations and simplifications, the stretching and bending forces are found, respectively, as

(7) $$\begin{array}{c} {\vec{F}}_{s}=\frac{\partial }{\partial s}\left(T\vec{\tau }\right),\;\;{\vec{F}}_{b}=-\frac{\partial^{2}}{\partial s^{2}}\left(c_{b}\frac{\partial^{2}\vec{X}}{\partial s^{2}}\right). \end{array}$$

Here, *T* is the tension and $\vec{\tau }$ is the unit tangent to the boundary. These are defined as

(8) $$\begin{array}{c} T=\{\begin{array}{cc} c_{s}\left(\left|\frac{\partial \vec{X}}{\partial s}\right|-1\right) & \left|\frac{\partial \vec{X}}{\partial s}\right|\geq 1\\ 0 & \text{otherwise}, \end{array}\\ \vec{\tau }=\frac{\partial \vec{X}/\partial s}{\left|\partial \vec{X}/\partial s\right|}, \end{array}$$

where the stiffness coefficient *c*_*s*_ is assumed to be uniform along the immersed boundary length. The larger *c*_*s*_ is, the stiffer the elastic boundary is and the greater force is induced by a stretching of the boundary.

It may be noted that all the variables and forces described through (5)–(8) are in the Lagrangian form, which should be transformed to the Eulerian form in order to be used in the Stokes equations. A detailed discussion about this process will be given in the following section. It is worth mentioning that throughout the paper, the Lagrangian and Eulerian variables are stated in uppercase and lowercase letters, respectively.

### 2.2. Fluid-Structure Interaction

The transformation between the Eulerian and Lagrangian variables can be realized by the Dirac delta function [5]. Spreading of the Lagrangian forcing to the nearby Eulerian grid points is expressed as

(9) $$\begin{array}{c} \vec{f}\left(\vec{x},t\right)=\overset{l_{0}}{\underset{0}{\int }}\vec{F}\left(s,t\right)\delta \left(\vec{x}-\vec{X}\left(s,t\right)\right)ds, \end{array}$$

where *δ* denotes the Dirac delta function. In the present work, we use the delta function proposed in [24]:

(10) $$\begin{array}{c} \delta_{h}\left(\vec{x}\right)=\delta_{h}\left(x\right)\delta_{h}\left(y\right). \end{array}$$

In addition, the local fluid velocity at the immersed boundary position $\vec{X}$ is obtained by interpolating the fluid velocity as follows:

(11) $$\begin{array}{c} \vec{U}\left(s,t\right)=\vec{u}\left(\vec{X}\left(s,t\right),t\right)=\overset{}{\underset{\Omega }{\int }}\vec{u}\left(\vec{x},t\right)\delta \left(\vec{X}\left(s,t\right)-\vec{x}\right)d\vec{x}, \end{array}$$

where *Ω* denotes the fluid domain to be influenced by the motion of the immersed boundary. The immersed boundary position is then updated by using the following equation:

(12) $$\begin{array}{c} \frac{\partial \vec{X}\left(s,t\right)}{\partial t}=\vec{U}\left(s,t\right). \end{array}$$

## 3. Numerical Method

### 3.1. Discretization of the Interface Lagrangian Force

**Figure 1:**
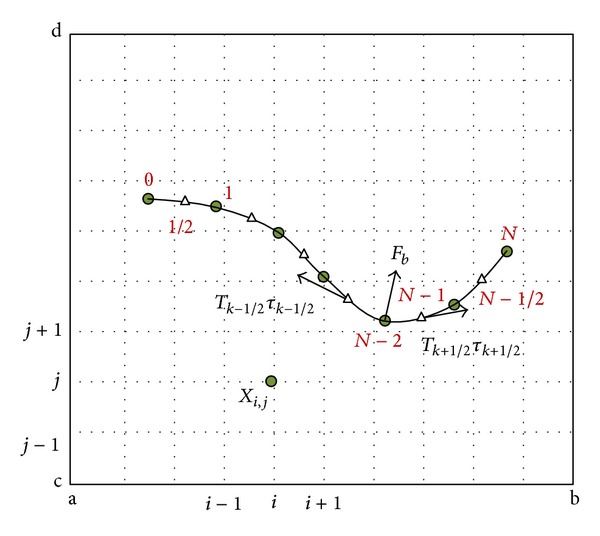
Schematic representation of the Lagrangian coordinate system for a flexible filament located in the Eulerian grids.

A staggered grid (marked nodal points) is used in the Lagrangian coordinate system (see Figure 1), where the tension forces are defined on the midpoints between the nodes and the bending forces are defined on the nodes. In the cases including filament, the numbering of nodes starts from the free end of the filament (*k* = 0) and ends at its fixed end (*k* = *N*).

Suppose that *D*_*s*_, *D*_*ss*_, and *D*_*sss*_ denote, respectively, the finite difference (FD) approximation to the first, second, and third order derivatives with respect to the arc-length *s*. For an arbitrary variable *ψ*, for instance, the following notations:

(13) $$\begin{array}{c} D_{s}^{0}\psi =\frac{\left(\psi \left(s+\Delta s/2\right)-\psi \left(s-\Delta s/2\right)\right)}{\Delta s},\\ D_{s}^{+}\psi =\frac{\left(\psi \left(s+\Delta s\right)-\psi \left(s\right)\right)}{\Delta s},\\ D_{s}^{-}\psi =\frac{\left(\psi \left(s\right)-\psi \left(s-\Delta s\right)\right)}{\Delta s}. \end{array}$$

Denote, respectively, the central, forward, and backward FD approximations of the first derivative. In addition, the second-order central FD approximation of the second derivative can be expressed as

(14) $$\begin{array}{c} D_{s}^{+}D_{s}^{-}\psi =\frac{\left(\psi \left(s+\Delta s\right)-2\psi \left(s\right)+\psi \left(s-\Delta s\right)\right)}{\Delta s^{2}}. \end{array}$$

The boundary tension *T*  and the tangent vector $\vec{\tau }$ (see (8)) can be discretized as

(15) $$\begin{array}{c} T_{k+1/2}=\{\begin{array}{cc} c_{s}\left(\left|D_{s}^{+}\vec{X}\right|-1\right) & \left|D_{s}^{+}\vec{X}\right|\geq 1\\ 0 & \text{otherwise}, \end{array}\\ {\vec{\tau }}_{k+1/2}=\frac{D_{s}^{+}\vec{X}}{\left|D_{s}^{+}\vec{X}\right|}. \end{array}$$

Finally, the discrete stretching force density ${\vec{F}}_{s}$ and the discrete bending force density ${\vec{F}}_{b}$ are given by

(16) (F→s)k=Tk+1/2τ→k+1/2−Tk−1/2τ→k−1/2Δs k=1,2,…,N−1,(F→b)k=−[Ds+Ds−(cbDssX→)]k=−cb(DssX→)k+1−2(DssX→)k+(DssX→)k−1Δs2k=1,2,…,N−1,

where,

(17) (DssX→)k={0k=0(Ds+Ds−X→)kk=1,2,…,N−1.

A clamped boundary condition is applied at the fixed end of the filament [25]:

(18) $$\begin{array}{c} {\left(D_{ss}\vec{X}\right)}_{N}=\frac{\left[\left(-1,0\right)-{\left(D_{s}^{0}\vec{X}\right)}_{N-1/2}\right]\;}{0.5\Delta s}. \end{array}$$

At the free end of the filament (*k* = 0), the bending force term is discretized as

(19) (F→b)0=−cb[Ds+(DsssX→)]0=−cb(DsssX→)1−(DsssX→)0Δs=−cb(Ds+Ds−X→)2−(Ds+Ds−X→)1Δs2.

### 3.2. Discretization of the Poisson Equation

It was mentioned in Section 2 that the Poisson equation governs both the velocity and pressure fields for the Stokes flow. In order to generalize the discretization scheme, the velocity and pressure variables are replaced by the general variable *φ* and the immersed boundary force is replaced by the variable *a*. Therefore, the general form of the Poisson equation can be written as follows:

(20) $$\begin{array}{c} \nabla^{2}\varphi =a_{i,j}. \end{array}$$

The fluid domain considered in the present work is a square box with periodic boundary conditions. The derivatives in (20) can be approximated by second-order central FD scheme as follows:

(21) 1Δx2(φi−1,j−2φi,j+φi+1,j) +1Δy2(φi,j−1−2φi,j+φi,j+1)=ai,j.

The periodic boundary conditions would imply that

(22) $$\begin{array}{c} \varphi_{i,j}=\varphi_{i+M,j+N}\;\forall i,j. \end{array}$$

Equation (21) along with the periodic boundary conditions (22) constitutes a system of linear equations. To solve this system of equations we use a fast direct solution method, which takes the advantage of the cyclic reduction algorithm. The cyclic reduction algorithm is a recursive scheme that eliminates half of the unknowns at each step until there remains a single equation that can be solved. The remaining unknowns are computed easily by a back-substitution method (for more information about this algorithm one may refer to [26–28]).

### 3.3. Discretization of FSI Equations

We now consider the equations connecting the fluid lattice and the immersed boundary points. Since the positions of the immersed boundary points generally do not coincide with those of the lattice points, we have to interpolate the velocity field from the fluid lattice to these points and spread the Lagrangian force from the immersed boundary points to the nearby lattice points of the fluid. This can be done by introducing a sufficiently smooth approximation to the Dirac delta function:

(23) $$\begin{array}{c} \delta_{h}\left(\overset{⇀}{x}\right)=\delta_{h}\left(x_{1}\right)\delta_{h}\left(x_{2}\right). \end{array}$$

The following discrete delta function is used to give the second-order approximations:

(24) $$\begin{array}{c} \delta \left(x\right)=\{\begin{array}{cc} \frac{1}{4\pi }\left[\pi +2\sin\left(\frac{\pi }{4}\left(2x+1\right)\right)-2\sin\left(\frac{\pi }{4}\left(2x-1\right)\right)\right] & \left|x\right|\leq 1.5\\ -\frac{1}{8\pi }\left[-5\pi +2\pi \left|x\right|+4\sin\left(\frac{\pi }{4}\left(2\left|x\right|-1\right)\right)\right] & 1.5\leq \left|x\right|\leq 2.5\\ 0 & 2.5\leq \left|x\right|. \end{array} \end{array}$$

It is worth noting that the above *δ* function covers wider support points as compared with that used by Peskin and McQueen [4]. This can effectively reduce the nonphysical oscillations (if any). More reasons for this particular choice of *δ* function are given in [24].

The discretized form of the local fluid velocity at the structure position $\vec{X}$ can be written as

(25) $$\begin{array}{c} U_{k}=\overset{N}{\underset{i,j=0}{\sum }}u_{i,j}\delta_{h}\left(x_{i,j}-X_{k}\right)h^{2}, \end{array}$$

where *h* is the Eulerian grid spacing. Similarly, the discretized form of the force density is given by

(26) $$\begin{array}{c} f_{i,j}=\overset{N_{b}-1}{\underset{k=0}{\sum }}F_{k}\left(X_{0},X_{1},\ldots ,X_{N_{b}-1}\right)\delta_{h}\left(x_{i,j}-X_{k}\right)\Delta s. \end{array}$$

Finally, the kinematic boundary condition (12), which is used to update the immersed boundary points in the fluid domain, is discretized using explicit forward Euler scheme:

(27) $$\begin{array}{c} X_{k}^{n+1}=X_{k}^{n}+\Delta tU_{k}^{n},\\ Y_{k}^{n+1}=Y_{k}^{n}+\Delta tV_{k}^{n}. \end{array}$$

### 3.4. Solution Algorithm

The numerical algorithm used in the present work for simulating the fluid-structure interactions as well as the fluid-mediated structure-structure interactions can be summarized as follows.

1. Compute Lagrangian forces *F* ^*n*^ at Lagrangian points *X* ^*n*^ using (5).
2. Communicate Lagrangian forces *F* ^*n*^ to Eulerian grid points using (9).
3. Solve fluid equations from (1) to (3) to get new velocity *u*.
4. Find the local fluid velocity at the immersed boundary position *U* using (11).
5. Update immersed boundary position using *U* and (12).

## 4. Results and Discussion

### 4.1. Flow Induced by a Relaxing Star String

In this test case, we study the motion of a nonequilibrium star string immersed in an incompressible fluid while relaxing to its circular equilibrium shape. The initial velocity and pressure of the fluid are set to zero, and the only driving force is the string tension. The fluid flow and the string motion are fully coupled. At equilibrium, the velocity is zero and the pressure is piecewise constant inside and outside the balloon. The initial shape of the distorted string is expressed in the cylindrical coordinates as

(28) $$\begin{array}{c} \rho =0.6+0.3\sin8\theta . \end{array}$$

**Figure 2:**
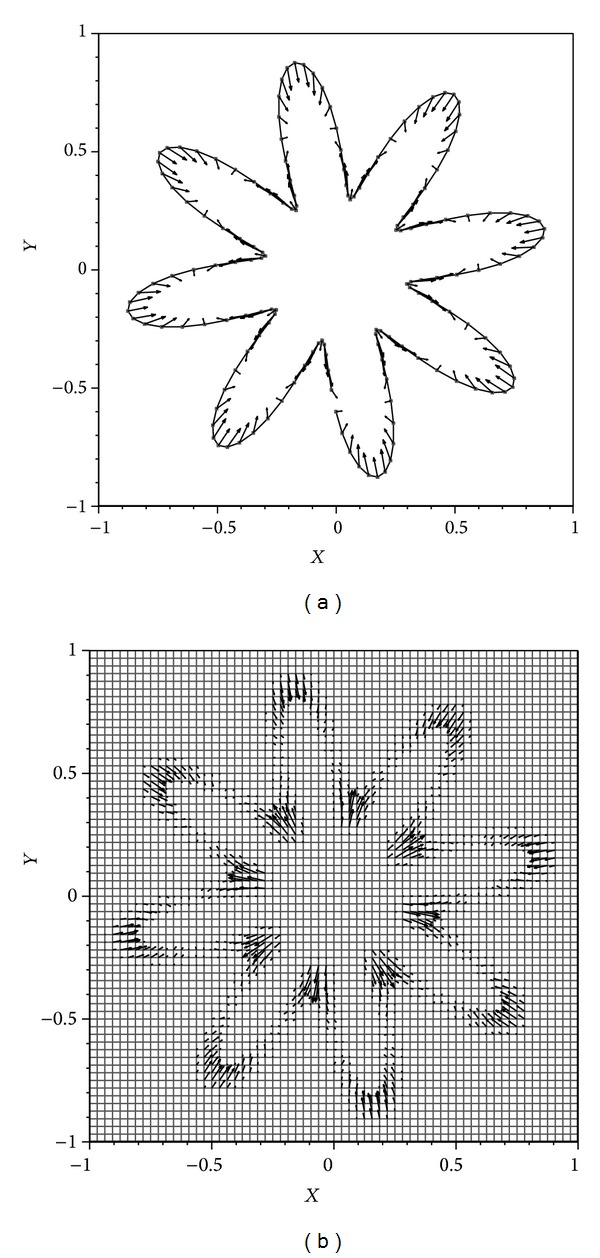
(a) Lagrangian forces associated with the elastic boundary at *t* = 0; (b) Eulerian force associated with the fluid domain at *t* = 0.

**Figure 3:**
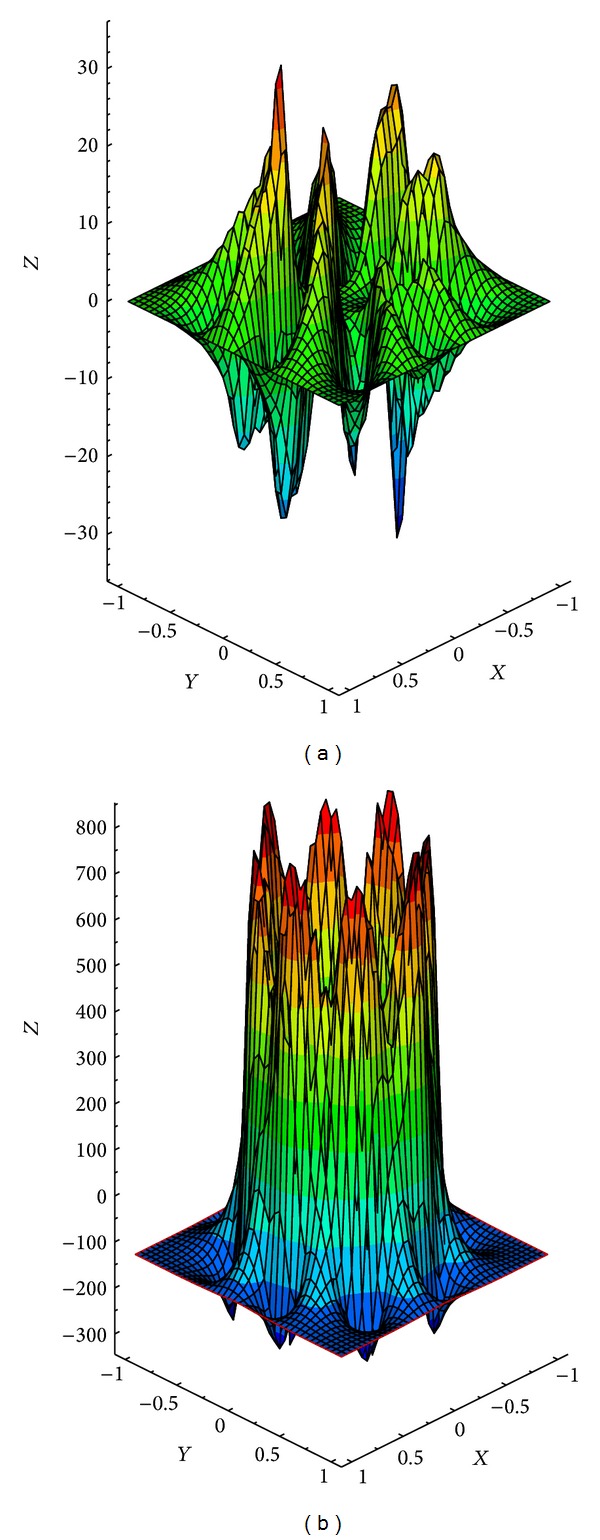
Velocity and pressure fields at time *t* = 0. With *μ* = 1, stiffness efficient *c*_*s*_ = 100: (a) *x*-component velocity field; (b) pressure field.

The unstretched interface is the circle with the radius *r*_0_ = 0.3. The problem is very stiff and we need to take a fairly small time step even with the implicit method. We started with Δ*t* = *O*(*h* ^2^) but could increase the time step at later times. Starting the work we compute velocity, pressure, Lagrangian, and Eulerian forces at the first time step. A grid size of 64 × 64 in a square domain with the dimensions of (−1,1)×(−1,1) is considered. The results have been shown in Figures 2 and 3.  Figure 3(b) shows the jumps in the pressure and the consequent discontinuity at the immersed boundary.

**Figure 4:**
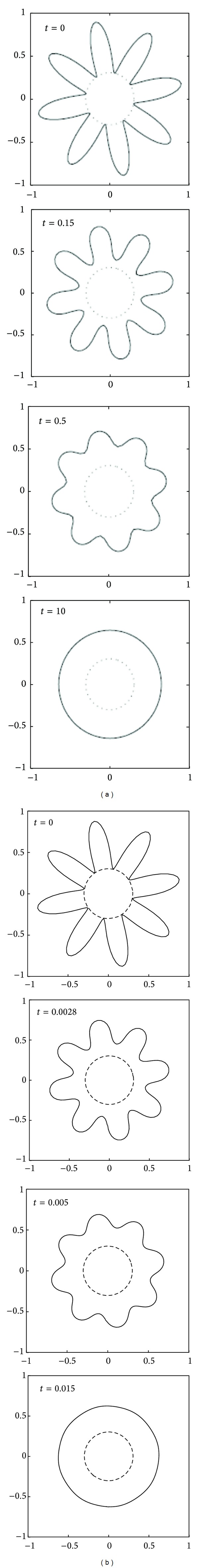
Interface of the star balloon at different times. The dotted circle represents the unstretched interface with *r* = 0.3 (a): IIM and (b): IBM.

Figure 4 shows the location of the interface at several times that agree quite well with the results of LeVeque and Li [19] using IIM method. The discrepancies in the times may be attributed partly to the fact that different stiffness coefficients have been used in the two methods.

It can be seen from Figure 4 that the immersed boundary moves in the fluid for some period of time and then attains its equilibrium state. At the equilibrium state, the pressure in the fluid is balanced with the stretching force of the boundary. At this state, the pressure inside the boundary is homogeneous and therefore it takes the circular shape.

**Figure 5:**
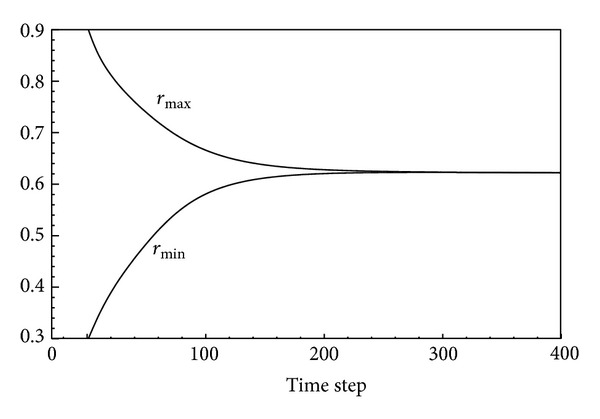
Plot of *r*_max⁡_ (upper curve) and *r*_min⁡_ (lower curve) showing the distance from the origin to the interface as a function of time *t* on a 64 × 64 grid with *N*_*b*_ = 160 on the boundary. The maximum and minimum radii will converge to the radius of a near-circle shape.

According to Figure 5, the star starts moving from its nonequilibrium state with the maximum and minimum radii of 0.9 and 0.3, respectively, and its radii converge to the radius 0.63 of a circle, which corresponds to the equilibrium state of the boundary. This figure shows how *r*_min⁡_^*n*^ and *r*_max⁡_^*n*^ behave computationally over a short-time scale.

The instantaneous maximum and minimum radii of the boundary are obtained from the following relations:

(29) rmin⁡n=min⁡(Xkn)2+(Ykn)2rmax⁡n=max⁡(Xkn)2+(Ykn)21≤k≤Nb.

### 4.2. Bending Filament

**Figure 6:**
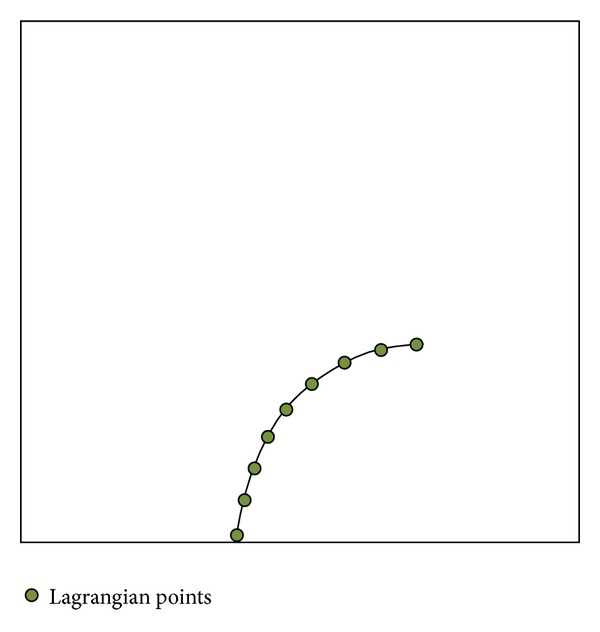
Initial geometry of the filament showing Lagrangian points.

In this section, the Stokes flow induced by the motion of a bending filament is examined. An initial bending force is applied to the filament. Initially the fluid is at rest. After the first time step, the Lagrangian points representing the filament boundary move from their initial positions in the flow direction. This leads the points to become too close to (or too far away from) each other. To keep the inextensibility of the filament, a (relatively) large stiffness coefficient is applied. Under such conditions, only the angle between the nodes varies, but the distance between them remains unchanged. In addition, to circumvent the instability arising when proper tension and bending forces are not applied, very small time steps are considered. As shown in Figure 6, the initial shape of the filament is considered as the arc of a circle obtained from the following relation:

(30) $$\begin{array}{c} \vec{X}\left(\theta \right)=\left(\begin{array}{c} r\cos\theta \\ \left|r\sin\theta \right| \end{array}\right)-\left(\begin{array}{c} r\\ 0 \end{array}\right)\;0\leq \theta \leq \frac{\pi }{2}. \end{array}$$

**Figure 7:**
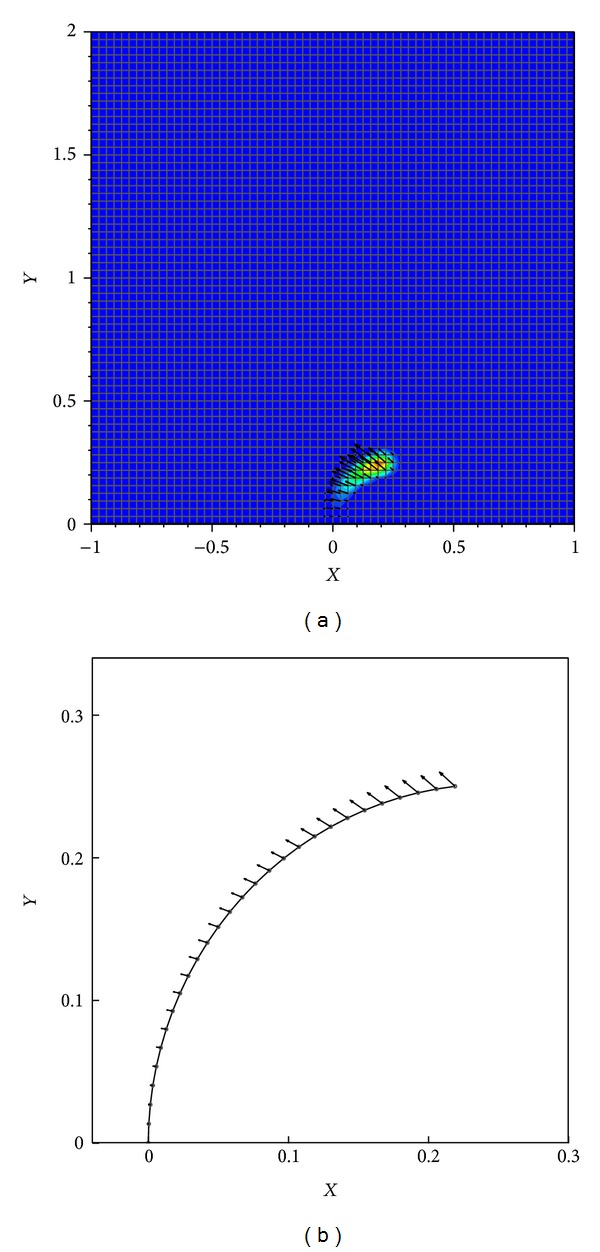
Eulerian (a) and Lagrangian (b) forces at *t* = 0.

The filament is clamped to the bottom wall of a square cavity. As mentioned above, initially the force applied by the elastic boundary on the fluid domain is due only to the bending forces. The tension forces are then added to the bending forces at the next time steps. The Lagrangian forces associated with the bending filament as well as the Eulerian forces applied on the fluid domain at the time *t* = 0 are depicted in Figure 7.

By inserting the Eulerian forces to the flow field equations and solving them, the velocity and pressure fields can be obtained at different time steps. The values of various constants considered for the case of the bending filament are given in Table 1.

**Figure 8:**
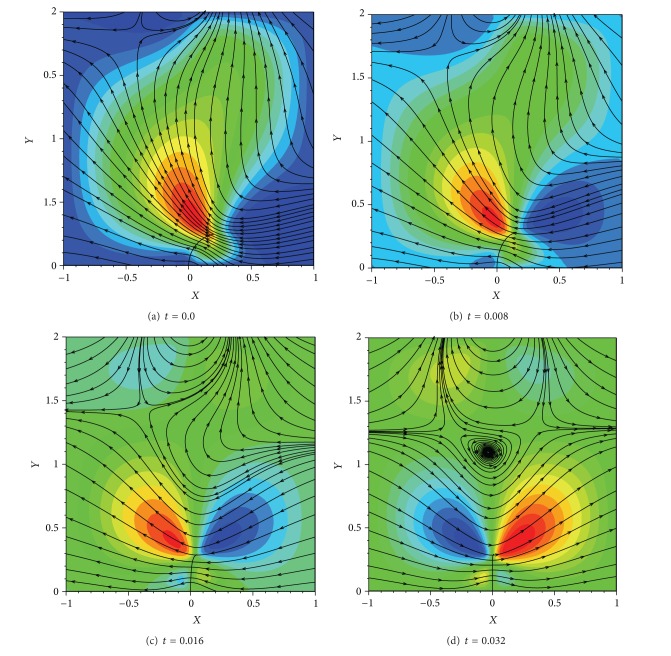
Contours of *x*-component velocity along with streamlines at different times of 0.0, 0.008, 0.016, and 0.032 corresponding to the frames, (a), (b), (c), and (d), respectively.

Figure 8 depicts the *x*-component velocity along with the streamlines at different time steps. It can be seen that the motion of the filament from its initial nonequilibrium state (Frame (a) in Figure 8) induces the fluid motion in the direction shown by the streamlines. Frame (d) in Figure 8 shows that at the time step of 0.032 the filament has slightly passed its equilibrium state to the left. Therefore, it is again in nonequilibrium state and has to return to the equilibrium state. During this process, it pushes the fluid in the opposite direction of the frames (a), (b), and (c). In addition, an eddy appears to be formed in the fluid domain above the filament.

**Figure 9:**
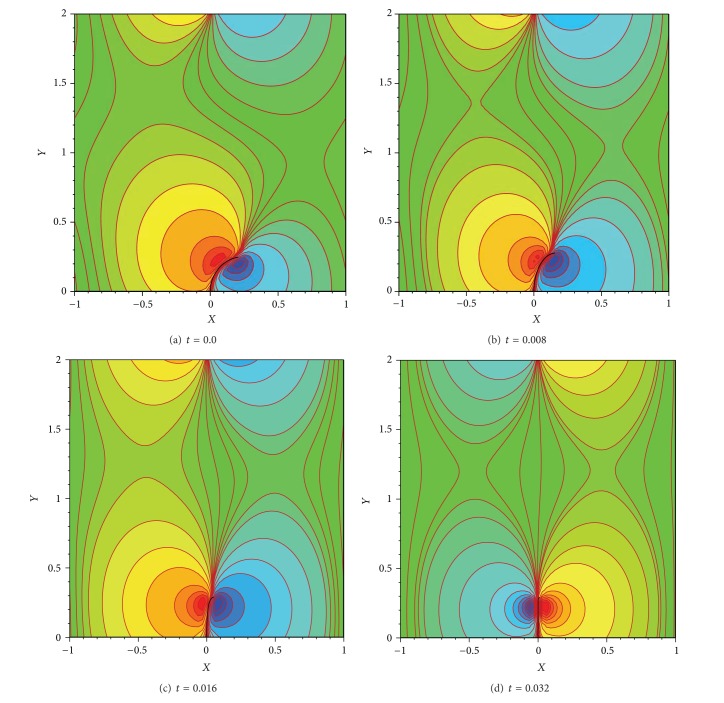
Pressure contours at different time steps of 0.0, 0.008, 0.016, and 0.032 corresponding to the frames (a), (b), (c), and (d), respectively.

The pressure contours associated with the different times shown in Figure 8 are plotted in Figure 9. According to this figure, the motion of the filament from its nonequilibrium state leads to a pressure variation around it. Due to the motion of the bending filament from right to left, vacuum is observed in the fluid located on the right side of the filament and hence minimum pressure is observed in this region. In contrast, it pressurizes the fluid on its left side and a maximum pressure is seen. This implies clearly the pressure jump and/or discontinuity on the immersed boundary.

### 4.3. Fluid-Mediated Structure-Structure Interaction

In this last test case, the motion of an initially bent filament in a square cavity causes the fluid motion, which in turn leads to the motion and deformation of an initially circular elastic boundary placed somewhere in the cavity. It may be noted that, for an immersed boundary to move in a fluid, either the fluid should have enough velocity to move the boundary or the initial deformation of the boundary itself would cause the fluid motion. In the present example, both of these mechanisms are present simultaneously.

**Figure 10:**
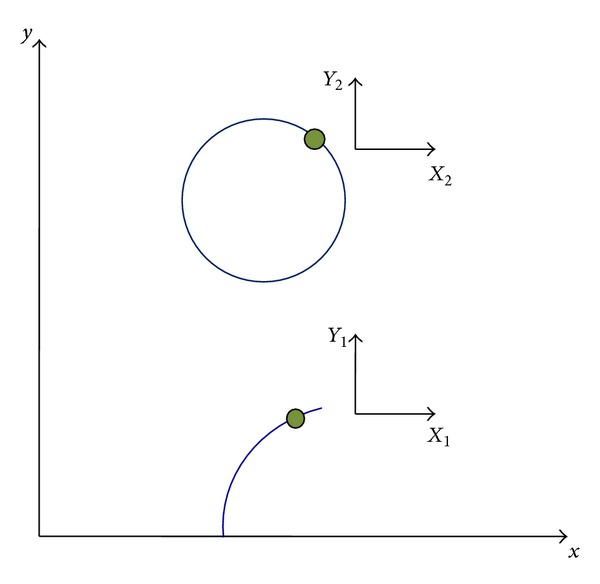
Lagrangian coordinate systems defined in the Eulerian solution domain.

The secondary immersed boundary considered here is an elastic circular string, which is initially in its equilibrium state without any deformation or internal forces. For this boundary, the tension forces are applied to avoid the Lagrangian points representing the boundary to move too far from (or too close to) each other. In addition, no bending force has been applied to this boundary in order to allow the angle between the points to change freely. As shown in Figure 10, in order to define both of the immersed boundaries (i.e., the filament and the circle), two distinct local Lagrangian coordinate systems are required to be defined in the Eulerian solution domain. The initial configurations of the filament and the circle can be obtained from relations (30) and (31), respectively:

(31) $$\begin{array}{c} \vec{X}\left(\theta \right)=\left(\begin{array}{c} 0.3\cos\theta \\ 0.3\sin\theta \end{array}\right)+\left(\begin{array}{c} 0\\ 1 \end{array}\right)\;0\leq \theta \leq 2\pi . \end{array}$$

**Figure 11:**
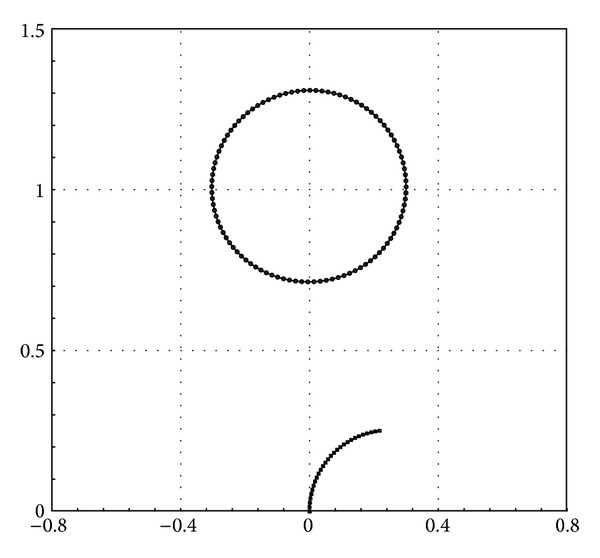
Initial configurations of the flexible boundaries immersed in the square flow domain.

The fluid domain (i.e., the square cavity) along with the initial configurations of the two immersed boundaries (i.e., a filament and a circular flexible boundary) is depicted in Figure 11. The values of constants used in this test case are given in Table 2. It is worth mentioning that the indices 1 and 2 are associated with the filament and circular boundary, respectively.

**Figure 12:**
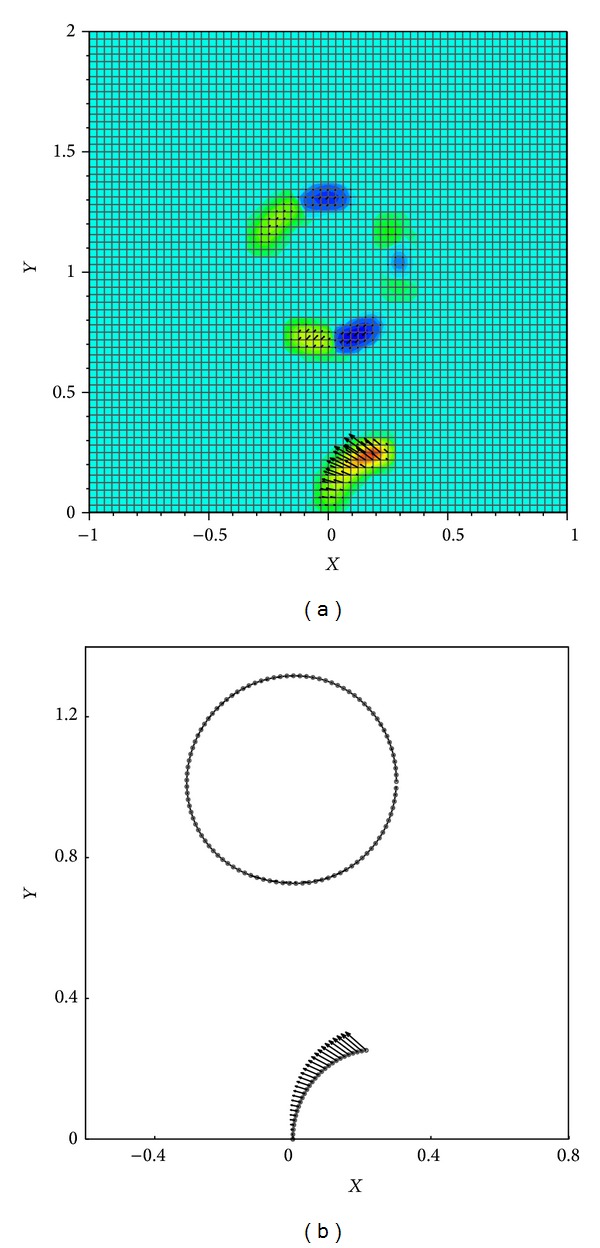
Eulerian forces imposed by the immersed boundaries on the fluid (a) and Lagrangian forces associated with the immersed boundaries (b) at time *t* = 0.001.

For the circular-immersed boundary, the stiffness coefficient is set to be relatively small to allow the points to move easily far away from each other. At the outset of the calculations, this boundary exerts no force on the initially quiescent fluid domain. In fact, the fluid motion is induced by the motion of the filament, which is initially bent. The moving fluid can now exert forces on the circular boundary and move and deform it. The Lagrangian forces associated with the immersed boundaries and the consequent Eulerian forces imposed on the fluid domain by theses boundaries at time *t* = 0.001 have been depicted in Figure 12.

**Figure 13:**
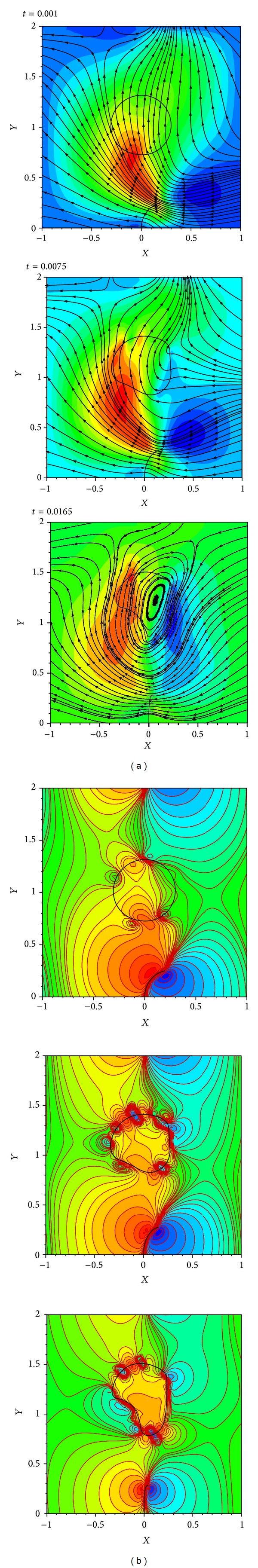
Contours of *x*-component velocity along with streamlines (a) and contours of pressure (b) at times 0.001, 0.0075, and 0.0165.

Figure 13 demonstrates the contours of *x*-component velocity along with the streamlines on the one hand and the contours of pressure on the other hand at times *t* = 0.001, 0.0075, and 0.0165. The sequential deformations of the immersed boundaries are also included in this figure. The circular boundary is found to experience significant deformation as it moves upward (i.e., towards the top boundary of the square domain). A single eddy forms inside the circle at time 0.0165. As expected, one can see from this figure that the pressure is discontinuous across both of the immersed boundaries.

## 5. Conclusions

In the present work, the Stokes flow induced by the motion of an elastic massless body immersed in a two-dimensional fluid is studied using immersed boundary method. Initially, the immersed body is unstable and the fluid is at rest. The body can induce fluid motion while returning to its equilibrium (stable) state leading to a fluid-structure interaction problem. In the current study, two different test cases are examined. In both of the cases, the motion of a massless filament fixed at one end induces the fluid motion inside a square domain. However, in the second test case, a deformable circular string is placed in the square domain and its interaction with the Stokes flow induced by the filament motion is studied. In order to verify the accuracy of the numerical method employed, the simulated results associated with the Stokes flow induced by the motion of an extending star string are compared well with those obtained by the immersed interface method. The results show the ability and accuracy of the IBM method in solving the complicated FSI problems happening in a wide variety of engineering and biological systems.

## Figures and Tables

**Table 1:** Values of constants associated with the case of bending filament.

Domain	(−1, 1) × (0, 2)
Number of Eulerian grid points	64 × 64
*N*_*b*_	30
*c*_*b*_	0.001
*c*_*s*_	2
Δ*t*	0.0004
μ	1
Initial radius	0.25

**Table 2:** Values of the constants associated with the filament and circular boundary.

Domain	(−1, 1) × (0, 2)
Number of Eulerian grid points	64 × 64
*N*_*b*_1__	30
*N*_*b*_2__	100
*c*_*s*_1__	2
*c*_*s*_2__	0.01
*c*_*b*_1,2__	0.001
μ	1
Δ*t*	0.0005
Initial radius 1	0.25
Initial radius 2	0.3
